# Anti‐Müllerian hormone type II receptor protein expression in non‐small cell lung cancer and the effect of AMH/AMHR2 signaling on cancer cell proliferation

**DOI:** 10.1111/1759-7714.15309

**Published:** 2024-09-04

**Authors:** Yoshika Koinuma, Yoichiro Mitsuishi, Wira Winardi, Moulid Hidayat, Aditya Wirawan, Daisuke Hayakawa, Koichiro Kanamori, Naohisa Matsumoto, Takuo Hayashi, Naoko Shimada, Ken Tajima, Kazuya Takamochi, Fumiyuki Takahashi, Kenji Suzuki, Kazuhisa Takahashi

**Affiliations:** ^1^ Department of Respiratory Medicine Juntendo University, Graduate School of Medicine Tokyo Japan; ^2^ Research Institute for Diseases of Old Ages Juntendo University, Graduate School of Medicine Tokyo Japan; ^3^ Department of Human Pathology Juntendo University, Graduate School of Medicine Tokyo Japan; ^4^ Department of General Thoracic Surgery Juntendo University, Graduate School of Medicine Tokyo Japan

**Keywords:** AMH‐AMHR2 pathway, cell cycle, cell proliferation, non‐small cell lung cancer

## Abstract

**Background:**

Non‐small cell lung cancer (NSCLC) is the leading cause of cancer‐related deaths worldwide despite advances in cancer therapeutics. In several gynecological cancers, anti‐Müllerian hormone receptor type 2 (AMHR2) mediates AMH‐induced growth inhibition and is expressed at high levels. Furthermore, 5%–8% of NSCLCs exhibit high AMHR2 expression, suggesting that AMH may inhibit the progression of some lung cancers. However, the clinical relevance of AMHR2 expression and its role in lung cancer is not fully clarified.

**Methods:**

Immunostaining was performed on 79 surgical specimens of NSCLC. The Cancer Genome Atlas RNA‐seq data for lung adenocarcinoma were analyzed, and gene ontology and gene set enrichment analyses were performed. In cellular experiments, AMHR2‐overexpressing NSCLC cell lines were established, and the role of the AMH‐AMHR2 pathway in cell proliferation with recombinant human AMH protein treatment was examined.

**Results:**

A total of 13 cases (16.5%) were positive for immunostaining in lung adenocarcinoma tissues; no positive signals were detected in lung squamous carcinoma tissues. Gene expression variation analysis using The Cancer Genome Atlas data showed that the expression of genes related to the cell cycle was downregulated in the AMHR2‐high group. Cellular experiments showed that activation of the AMH‐AMHR2 pathway suppressed cell proliferation.

**Conclusion:**

In lung adenocarcinoma tissues with high expression of AMHR2, activation of the AMH‐AMHR2 pathway may suppress cell proliferation.

## Graphical abstract

·Some lung adenocarcinoma clinical specimens express the AMHR2 protein. In lung adenocarcinoma tissues with high AMHR2 expression, activation of the AMH–AMHR2 pathway may suppress cell proliferation.
·This study unveils potential therapeutic targets for future research by suggesting that the AMH–AMHR2 pathway plays a role in suppressing cancer progression in cases of lung adenocarcinoma with high AMHR2 expression.

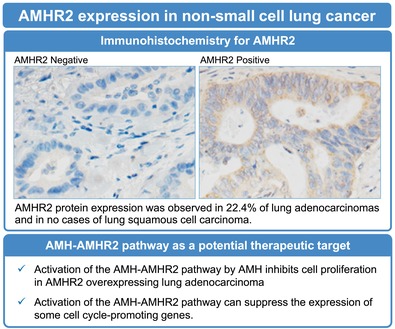

## INTRODUCTION

Lung cancer is one of the most common cancers worldwide, leading to both high incidence and mortality.^1^ Recent research has shown that subsets of non‐small cell lung cancer (NSCLC) can be distinguished based on recurrent driver mutations in different oncogenes.^2^ The advent of molecular targeted therapies, particularly those targeting receptor tyrosine kinases in advanced NSCLC (e.g., mutations in the epidermal growth factor receptor [*EGFR*] or anaplastic lymphoma kinase [*ALK*] genes), has revolutionized lung cancer treatment. Although the introduction of these targeted drugs along with recent developments in immune checkpoint inhibitors has significantly improved the prognosis of lung cancer, overall patient survival rates remain suboptimal.^3^, ^4^ This underscores the importance of expanding our understanding of effective treatment mechanisms to further improve patient outcomes.

Anti‐Müllerian hormone (AMH), also known as Müllerian‐inhibiting substance (MIS), is a 560‐amino acid (including a signal peptide) glycoprotein that belongs to the transforming growth factor beta superfamily.^5^, ^6^ AMH is essential for the induction of Müllerian duct regression in male embryos.^7^ It is synthesized in both male and female gonads by Sertoli cells in the testes and by granulosa cells in the ovaries.^8^ AMH binds to the AMHR2 receptor located on cells within the developing gonads. Studies have shown that AMH not only plays a critical role in embryonic sexual differentiation but also inhibits the growth of cells with AMH receptors. AMH binds to a heterodimeric receptor composed of AMH receptor types 1 and 2 (AMHR1 and AMHR2, respectively).^9^, ^10^, ^11^ Specifically, AMHR2 is the key player in mediating AMH‐induced growth inhibition^6^ and has been reported to be expressed at high levels in some cancer types.^12^, ^13^, ^14^ As AMH has been shown to inhibit tumor cell proliferation in several gynecological cancers, including ovarian cancer, through AMHR2‐mediated actions, the AMH‐AMHR2 signaling pathway has emerged as a promising therapeutic target for ovarian cancer.^15^, ^16^, ^17^

The expression of AMHR2 in nongynecological cancers has rarely been reported. A recent screening for AMHR2 protein expression using a panel of 631 samples from 15 different nongynecological cancers (including 18 NSCLC cases) revealed that AMHR2 is expressed in various solid tumors. It is particularly abundant in renal cell carcinoma, colorectal cancer, and liver cancer.^13^ An analysis of transcriptomic data from The Cancer Genome Atlas (TCGA) for NSCLC showed that AMHR2 is highly expressed at the mRNA level in 6% of lung adenocarcinomas and 8% of squamous cell carcinomas.^18^, ^19^ However, little is known about the extent of AMHR2 protein expression in NSCLC, the implications of its high expression, and the therapeutic potential of targeting its relevant pathway. Therefore, in this study, we aimed to validate the protein expression of AMHR2 in NSCLC using surgical specimens. We also investigated the therapeutic effect of AMH on NSCLC cell lines with high AMHR2 expression.

## METHODS

### Patients and tissue specimens

A total of 103 specimens of NSCLC were obtained from patients who underwent surgical resection between February 23, 2010, and February 2, 2011, at the Department of Surgery, Juntendo University Hospital, Tokyo, Japan. All the specimens were fixed in 10% formalin and embedded in paraffin wax. Data collected from the patients included age, sex, smoking index, TNM stage,^20^, ^21^ tumor size, lymph node metastasis, and histological type. This study was approved by the Institutional Review Board of Juntendo University Graduate School of Medicine (approval no.: 2017132). Informed consent was obtained from all patients enrolled in this study.

### Cell lines

The cell lines were obtained from the Cancer Cell Line Encyclopedia Project in 2016 and 2017.^22^ We performed short tandem repeat profiling of the cell lines to ensure authenticity and confirm the absence of contamination. Cells were routinely confirmed negative for mycoplasma contamination using a MycoAlert mycoplasma detection kit (LT07‐318; Lonza). The cells were maintained in RPMI‐1640 medium supplemented with 10% heat‐inactivated fetal calf serum (FCS) and 1% penicillin–streptomycin. The cells were passaged every 3–4 days.

### Immunohistochemistry

Tissues were fixed in 10% paraformaldehyde, embedded in paraffin, and cut into 4 μm sections. The sections were dehydrated and rehydrated in a graded series of ethanol solutions. After deparaffinization, the sections were subjected to antigen retrieval by heating in an autoclave at 120°C for 10 min in 0.01 M sodium citrate (pH 6.0) and were exposed to methanol with 2% H_2_O_2_, followed by washing in phosphate‐buffered saline with 0.05% Tween 20 (PBS‐T). The sections were then incubated with rabbit polyclonal antibody against AMHR2 (ab‐197 148; Abcam) in DAKO REAL antibody diluent (Dako) at 4°C overnight (approximately 0.5% dilution). After washing with PBS‐T, the sections were sequentially incubated with a horseradish peroxidase (HRP)‐polymer enzyme‐conjugated secondary antibody (N‐Histofine Simple Stain Max PO multi; Nichirei Biosciences Inc.) for 45 min at room temperature (20–25°C), washed with PBS‐T and PBS, and transferred directly to the chromogenic detection step using 3,3′‐diaminobenzidine. After 2 min of exposure, the sections were counterstained with hematoxylin.^23^

### RNA‐sequencing data and bioinformatic analysis

Transcriptomic data for 515 lung adenocarcinoma cases were obtained from TCGA using the MD Anderson Cancer Center MBatch Omic Browser (https://bioinformatics.mdanderson.org/MQA/). The cases were grouped according to AMHR2 mRNA expression level: the top 25% (128 cases) in the AMHR2‐high group, and the bottom 75% (387 cases) in the AMHR2‐low group. Differential gene expression analysis was performed using the DESEQ2 pipeline.^24^ Differentially expressed genes (DEGs) were subjected to gene ontology (GO) and Kyoto Encyclopedia of Gene and Genome (KEGG) enrichment analyses using g: Profiler, a public web server (http://biit.cs.ut.ee/gprofiler/).^25^, ^26^ Gene set enrichment analysis (GSEA) was performed using the Gene Pattern pipeline.^27^

### AMHR2 overexpression experiment

To establish AMHR2‐overexpressing cell lines, A549 and H1299 cells were purchased from the Riken Bioresource Center (Tokyo, Japan). pDONR223‐AMHR2 was gifted by William Hahn and David Root (Addgene Plasmid #23453). Gateway cloning was performed to insert AMHR2 into the pLEX_307 vector. Plasmids were cotransfected with psPAX2 and pMD2.G vectors into HEK‐293 T cells using the CalPhos Mammalian Transfection Kit (Takara Bio Inc.) to produce lentiviruses. The cells were infected with the virus and selected with 2 μg/mL puromycin.

### Immunoblotting

Immunoblotting was performed on A549 and H1299 cells, which overexpress AMHR2, and on both EGFP‐induced cell lines. Cells were cultured at 37°C in a 5% CO_2_ incubator under RPMI‐1640 containing 10% FCS. The cells were harvested and lysed for 30 min on ice in sodium dodecyl sulfate buffer containing a mixture of protease and phosphatase inhibitors (Roche). The lysate was centrifuged at 12000 × *g* for 15 min at 4°C, and the supernatant was collected and stored at −80°C. A DC protein assay was performed to determine protein concentration (Bio‐Rad Laboratories).

Blots were incubated overnight with primary antibodies for: AMHR2 (cat. no. ab197148; 1:1000; Abcam), GAPDH (cat. no. ab8245; 1:1000; Abcam), and V5 tag (cat. no. ab27671; 1:1000; Abcam) diluted in Can Get Signal Solution 1 (cat. no. NKB‐201; TOYOBO). Blots were then incubated overnight with the appropriate secondary antibodies (goat anti‐rabbit IgG H&L (HRP), ab6721, 1:5000, or rabbit antimouse IgG H&L (HRP), ab6728, 1:5000; Abcam) diluted in Can Get Signal Solution 2 (cat. no. NKB‐301, TOYOBO). The immunoreactive bands were visualized using can get signal immunoreaction enhancer solution (TOYOBO), and a ChemiDoc Touch MP imager (Bio‐Rad) was used to capture the western blot signals (Bio‐Rad).

### Cell proliferation assay with or without recombinant human AMH

Cell proliferation assays were performed on A549 and H1299 cells overexpressing AMHR compared with EGFP‐induced control cell lines. Cells were cultured in RPMI‐1640 containing 10% FCS at 37°C in a 5% CO_2_ incubator. The cells were plated in 96‐well microplates at a density of 1000 cells/well (100 μL/well). The 96‐well microplates were prepared for counting on days 0, 3, and 5 of the culture. Cell proliferation was evaluated using cell counting kit‐8 (CCK‐8; Dojindo). The 96‐well microplates on days 0 and 3 were evaluated without changing the medium after plating and on day 5 after changing the medium on day 3. For the cell proliferation assay using (recombinant human MIS/AMH protein [rhAMH] provided by R&D Systems), the cells were counted and plated in 96‐well microplates at a density of 1000 cells/well. After 1 day of incubation, the medium was changed to RPMI‐1640 with 1% FCS, and the following day, the medium was replaced with rhAMH with 1% FCS RPMI‐1640 medium. The cells were plated with or without rhAMH treatment at 20 and 50 ng/mL.

### RNA extraction, reverse transcription, and quantitative RT‐PCR

Quantitative RT‐PCR (qPCR) was performed to observe changes in gene expression between cell lines with and without rhAMH treatment. To extract RNA for qPCR, A549 cell lines with an EGFP‐induced control and those overexpressing AMHR were plated in 6‐cm dishes. After 1 day of incubation, the medium was changed to RPMI‐1640 with 1% FCS, and the following day, the medium was replaced with 1% FCS RPMI‐1640 medium again, with or without 50 ng/mL rhAMH. Total cellular RNA was extracted using the RNeasy Plus Mini Kit (Qiagen) according to the manufacturer's protocol. The purity and concentration of all RNA samples were quantified using a NanoDrop 2000 spectrophotometer (Thermo Fisher Scientific). A total of 500 ng of total RNA were reverse transcribed into cDNA using the Revertra Ace qPCR RT Kit (Toyobo). Quantitative PCR was performed using THUNDERBIRD Next SYBR qPCR Mix (Applied Biosystems). The cycling conditions were as follows: initial denaturation at 95°C for 20 s, followed by 40 cycles of amplification (denaturation at 95°C for 30 s, annealing and extension at 60°C for 30 s), and a final melting curve analysis. Quantitative PCR was performed in triplicate, and the expression level of β‐actin was used as an internal control. The primer sequences used for gene expression analysis by qPCR were as follows:

β‐actin
   Forward, 5'‐CACCATTGGCAATGAGCGGTTC‐3'
   Reverse, 5'‐AGGTCTTTGCGGATGTCCACGT‐3'
MCM4
   Forward, 5'‐CTTGCTTCAGCCTTGGCTCCAA‐3'
   Reverse, 5'‐GTCGCCACACAGCAAGATGTTG‐3'
MCM7
   Forward, 5'‐GCCAAGTCTCAGCTCCTGTCAT‐3'
   Reverse, 5'‐CCTCTAAGGTCAGTTCTCCACTC‐3'
PLK1
   Forward, 5'‐GCACAGTGTCAATGCCTCCAAG‐3'
   Reverse, 5'‐GCCGTACTTGTCCGAATAGTCC‐3'

### Statistical analysis

JMP 7 software was used to evaluate clinical data, and GraphPad Prism 9 was used to analyze TCGA and the results of the in vitro experiments. Welch's *t*‐test or Mann–Whitney U test was used for two‐group comparisons. Chi‐squared was used for categorical data. *p*‐values of <0.05 were considered statistically significant.

## RESULTS

### Immunohistochemical detection of AMHR2 in NSCLC tissues

**FIGURE 1:**
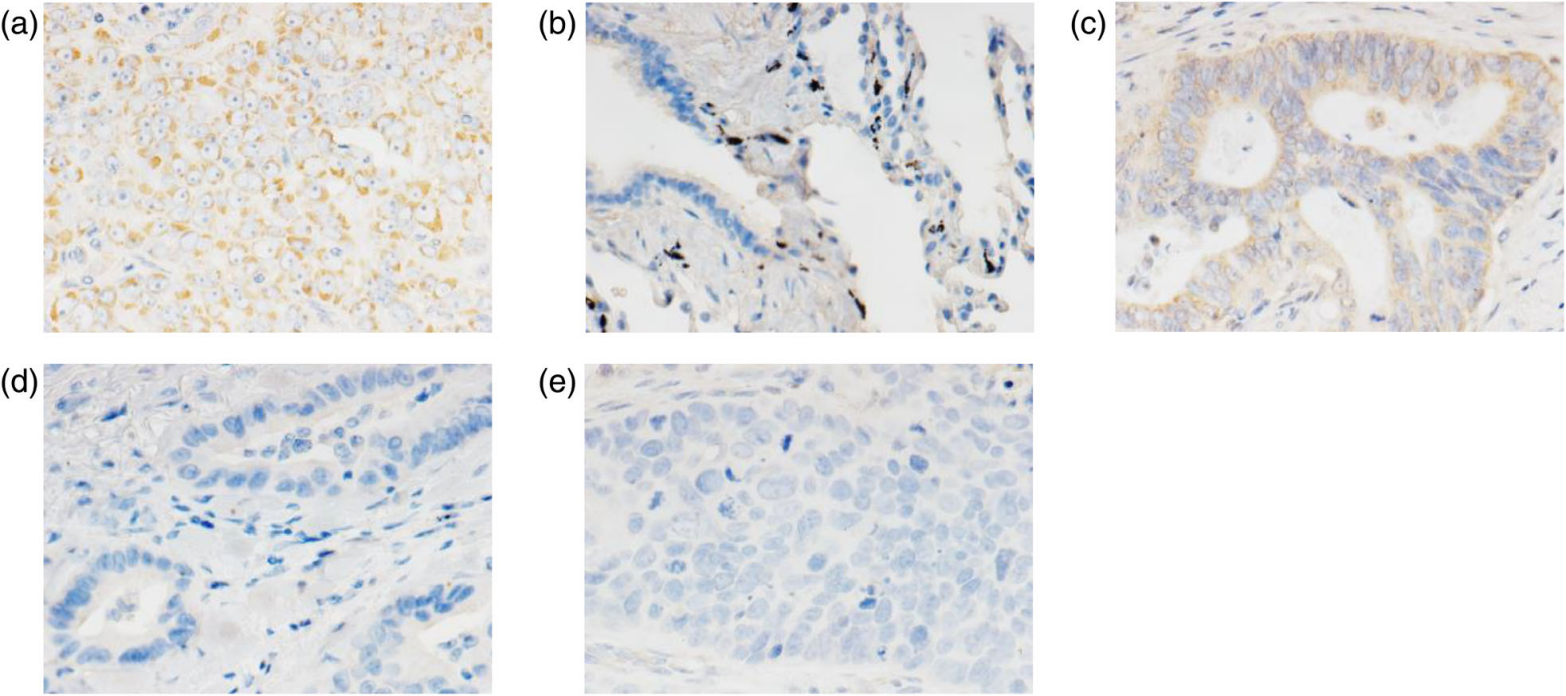
Immunohistochemical detection of AMHR2 in non‐small cell lung cancer and ovarian serous adenocarcinoma tissues. (a) Immunostaining of ovarian serous adenocarcinoma tissue. (b) Normal lung tissue. (c) Representative lung adenocarcinoma tissue showing positive staining for AMHR2. (d) Lung adenocarcinoma tissue showing negative staining for AMHR2. (e) Lung squamous cell carcinoma tissue. All micrographs were captured at 400× magnification.

According to the public resource of the Genotype‐Tissue Expression project for studying tissue‐specific gene expression, AMHR2 is highly expressed in the adrenal gland, ovary, and testis but not in normal lung tissue (Figure S1).^28^ In addition, previous reports have shown that AMHR2 expression is common in ovarian cancer cases.^12^, ^29^ Based on these findings, we first optimized the immunostaining conditions of AMHR2 using human ovarian serous adenocarcinoma tissue as a positive control.^16^ As shown in Figure 1a, AMHR2 expression was detected mainly in the cytoplasm of tumor cells with a granular pattern in the immunostaining of human ovarian serous adenocarcinoma tissues. In contrast, AMHR2 expression was not detected in the normal lung tissue (Figure 1b). These results indicate that AMHR2 expression can be adequately evaluated via immunohistochemical examination.

Under the same conditions, surgically resected cancer specimens from 79 patients with NSCLC were stained with anti‐AMHR2 antibody. AMHR2‐positive cases were defined as those in which signals were detected in the cytoplasm in a granular pattern (Figure 1c). Interestingly, positive signals were detected only in lung adenocarcinoma tissues and not in any of the squamous cell carcinoma tissues tested in this study. Of the 58 lung adenocarcinoma cases tested, 13 (22.4%) were positive for AMHR2. In terms of cellular localization, AMHR2 protein was detected at the membrane and in the cytoplasm.

### Association between AMHR2 expression and the clinicopathological features of patients with lung adenocarcinoma

The mean age of the patients was 63 years (range 43–86 years). The patients examined in this study did not receive radiation or chemotherapy prior to the surgery. The mean follow‐up time was 2195 days (range 229–3050 days). The samples included 58 lung adenocarcinomas and 28 lung squamous cell carcinomas. Owing to the lack of AMHR2‐positive cases in lung squamous cell carcinoma tissues, we investigated the association between AMHR2 immunoreactivity and the clinicopathological features of lung adenocarcinoma cases (Table 1). Although AMH/AMHR2 is known to be involved in sexual differentiation, and AMH secretion declines, at least in older age groups,^8^, ^30^ no significant difference between sex and age stratification of patients was observed. In addition to sex and age, no significant correlations were found between AMHR2 signaling, and the parameters examined, including smoking index, sex, pathological stage, and lymph node metastasis.

**TABLE 1:** Comparison of clinicopathological features between AMHR2‐positive and AMHR2‐negative patients with lung adenocarcinoma.

Characteristics	AMHR2 immunoreactivity		*p*‐value
	Positive (*n* = 13)	Negative (*n* = 45)	
Patient age (mean ± SD)	62 ± 7.4	68 ± 8.8	0.2199
Gender			
Male	6	29	0.9387
Female	7	16	
Smoking pack‐years (mean ± SD)	38 ± 39.8	34.74 ± 34.12	0.3424
TNM stage			
I	5	25	0.1575
II	2	7	
III	6	13	
Lymph node metastasis			
Positive	6	13	0.2170
Negative	7	32	
*EGFR* mutation status			
Positive	6	24	0.7516
Negative	7	21	
*KRAS* mutation status			
Positive	2	7	0.9387
Negative	11	38	

*Note*: Detailed comparison of various clinicopathological features among the 58 lung adenocarcinoma cases categorized based on AMHR2 immunoreactivity. Abbreviations: SD, standard deviation; TNM, tumor, node, metastasis. The Chi‐square test was used to determine significant differences between AMHR2‐positive and AMHR2‐negative groups.

### GO term and KEGG pathway enrichment analyses with AMHR2 overexpression in lung adenocarcinoma

**FIGURE 2:**
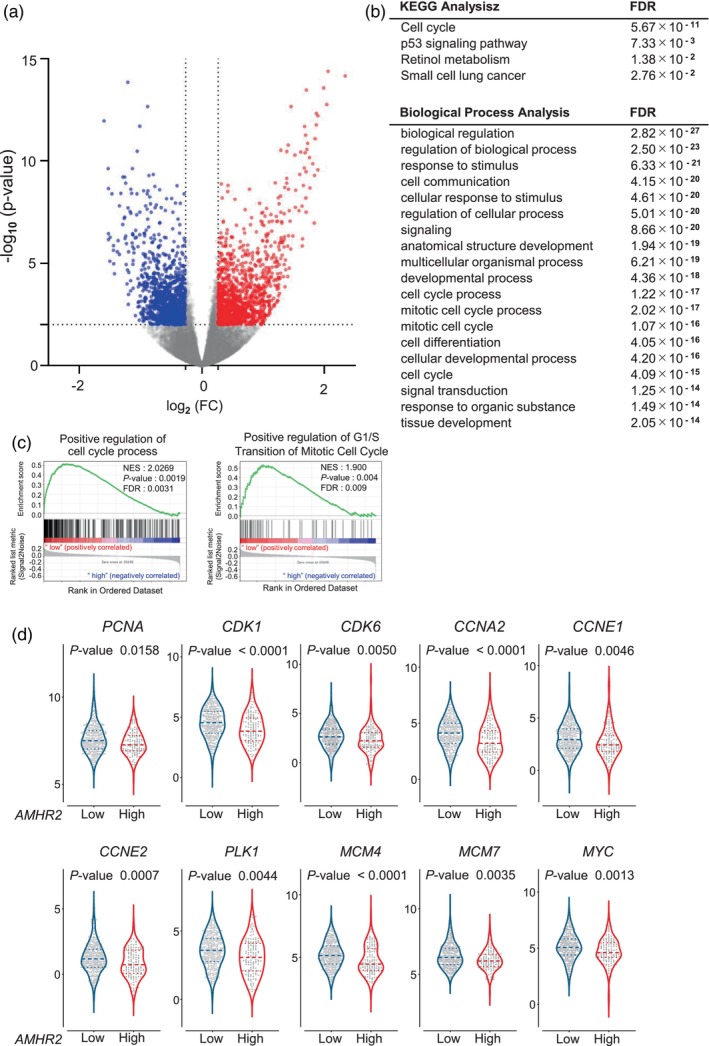
Differential gene expression analysis of The Cancer Genome Atlas (TCGA) RNA sequence data. (a) Volcano plot showing differential gene expression between the AMHR2‐high and AMHR2‐low groups among TCGA lung adenocarcinoma cases. Genes represented above the horizontal line are significantly differentially expressed with an FDR‐adjusted *p*‐value <0.01. Red dots represent genes with a fold‐change increase >1.2 in the AMHR2‐low group, indicating upregulation, while blue dots represent genes with a fold‐change decrease >1.2 in the AMHR2‐low group, indicating downregulation. (b) Pathway enrichment analysis of the genes significantly upregulated in the AMHR2‐low group. Results from both KEGG (upper section) and biological process (lower section) enrichment are shown. (c) Gene set enrichment analysis of RNA‐sequencing data from TCGA lung adenocarcinoma cases revealed an enrichment of cell cycle‐related genes in the AMHR2‐low group compared to the AMHR2‐high group. (d) Violin plots showing the expression of representative genes that respectively promote the cell cycle. Each dot is one sample. Red represents the AMHR2‐high group, and blue represents the AMHR2‐low group. Horizontal bars are presented as the 25th percentile (bottom quartile), median, and 75th percentile (top quartile). *p*‐values were obtained using the Mann–Whitney U test (a *p*‐value <0.05 was significant).

To explore the function of AMHR2 in lung adenocarcinoma, differential expression analysis was performed to identify DEGs between the AMHR2‐high and AMHR2‐low groups using the available TCGA RNA‐sequencing dataset. Analysis of the dataset from TCGA (*n* = 515) revealed that 1231 DEGs were significantly upregulated in the AMHR2‐low group and 1082 DEGs were downregulated in the AMHR2‐high group (Figure 2a).

To clarify the biological processes and molecular functions associated with our gene set, the upregulated DEGs were subjected to KEGG pathway enrichment analysis.^31^, ^32^, ^33^ The top enriched pathways included the “cell cycle” (*p*‐value = 4.90 × 10^−11^) and the “p53 pathway” (*p*‐value = 6.87 × 10^−3^) as significantly affected, suggesting that these genes play roles in the cell cycle and lung cancer progression. GO enrichment analysis performed using g:Profiler^25^, ^26^ identified several GO terms that were significantly associated with the gene set. Terms related to cellular functions and regulatory processes were also identified. In particular, “mitotic cell cycle process” (GO: 1903047; *p*‐value = 1.36 × 10^−17^) and “cell cycle process” (GO: 0022402; *p*‐value = 2.07 × 10^−17^) were among the significantly enriched terms. Other relevant terms included “biological regulation,” “regulation of cellular processes,” “response to stimulus”, “anatomical structure development,” and “cell communication,” highlighting the broad range of cellular and developmental processes affected by our gene set (Figure 2b). In the KEGG and GO analyses of downregulated DEGs, terms related to lipid metabolism were significantly enriched (Figure S4).

Given the potential association between AMHR2 expression and lung adenocarcinoma progression, GSEA was performed to determine whether there was a coordinated expression pattern between AMHR2‐related genes and established cell cycle gene sets. The results revealed a correlation between variations in AMHR2 expression and specific cellular processes (Figure 2c, Figure S5). Notably, gene sets associated with G1‐S phase transition and mitotic spindle assembly showed positive enrichment in samples with low AMHR2 expression, suggesting upregulation of these critical cell cycle phases (Figure 2c).

Interestingly, in the AMHR2‐low group, several key regulators known to drive cell cycle progression were concomitantly upregulated compared to those in the AMHR2‐high group. Specifically, key genes, such as *PCNA* (DNA replication marker), *CDK1* and *CDK6* (central cyclin‐dependent kinases), *CCNA2, CCNE1*, and *CCNE2* (cyclins regulating CDKs), *PLK1* (kinase involved in mitosis), *MCM7* and *MCM4* (members of the mini‐chromosome maintenance complex essential for DNA replication initiation), and *MYC* (transcription factor promoting cell cycle entry), were significantly upregulated (Figure 2d). This expression pattern suggests that the AMHR2‐low group may be predisposed to an accelerated cell cycle progression, resulting in cells likely to be more prone to rapid proliferation.

### Generation of non‐small cell lung cancer cell lines overexpressing AMHR2

AMH was initially investigated as a potential therapeutic candidate for various reproductive tract cancers because most reproductive tract tumors originate from tissues derived from the Müllerian duct and AMH plays an important role in causing Müllerian duct regression in male embryos.^15^, ^16^, ^17^ According to recent pan‐cancer analyses that provide a comprehensive characterization of over 11 000 tumors from 33 of the most common cancers, it has become more evident that AMHR2 is highly expressed in some tumors other than those of Müllerian duct origin, such as adrenocortical carcinoma, cutaneous melanoma, pheochromocytoma, malignant pleural mesothelioma, and lung adenocarcinoma (Figure S2). We hypothesized that the therapeutic effect of AMH may be applicable to lung adenocarcinomas with high AMHR2 expression.

**FIGURE 3:**
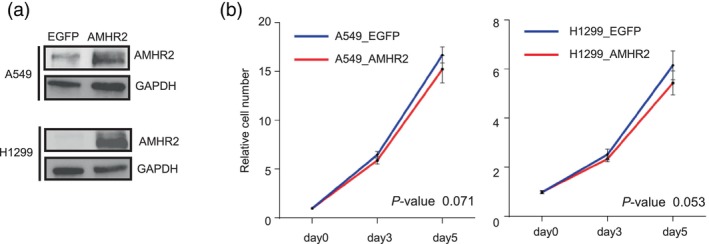
Generation of non‐small cell lung cancer cell lines overexpressing AMHR2 and their effect on proliferation. (a) Confirmation of AMHR2 overexpression in A549 and H1299 cell lines using western blot. (b) Growth comparison between AMHR2‐overexpressing and control A549 and H1299 cells. The y‐axis starts with day 0 mean values set to 1. Data from one representative of three independent experiments are shown, with error bars representing standard deviation. Statistical analysis was performed using Welch's *t*‐test.

Owing to the lack of NSCLC cell lines expressing high levels of AMHR2 (Figure S3), we established a model of AMHR2 overexpression in lung cancer cells using two NSCLC cell lines, A549 and H1299 (Figure 3a). The A549 and H1299 cells did not harbor any genetic mutations or copy number abnormalities in the *AMHR2* gene. As shown in Figure 3b, overexpression of AMHR2 slightly suppressed the proliferation of both cell lines.

### AMH inhibits growth of non‐small cell cancer cell lines expressing AMHR2

**FIGURE 4:**
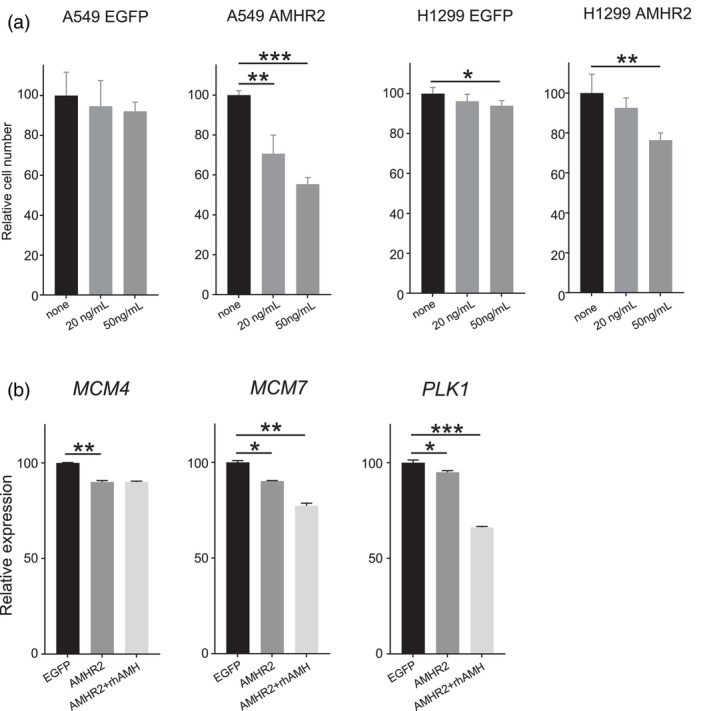
Effects of recombinant human AMH treatment on cell proliferation and cell cycle related gene expression in non‐small cell lung cancer cell lines overexpressing AMHR2. (a) Inhibitory effect of recombinant human AMH (rhAMH) on the proliferation of AMHR2‐overexpressing cell lines. Cell counts were obtained after 5 days of treatment with 20 or 50 ng/mL rhAMH. The y‐axis is normalized with control group mean values set to 100. Data from one representative of three independent experiments are shown, with error bars representing standard deviation. Statistical analysis was performed using Welch's *t*‐test. (*p < 0.05; **p < 0.01; ***p < 0.001). (b) Investigation of the effects of rhAMH on the genes using qPCR. The EGFP‐induced control A549 cell line (EGFP: left bar) as the control, AMHR2‐overexpressing A549 cell line (AMHR2: middle bar) and AMHR2‐overexpressing A549 cell line treated with 50 mg/mL rhAMH (AMHR2 + rhAMH: right bar) were compared. The qPCR was performed in triplicate, and data from one representative of six independent experiments are shown. The y‐axis is normalized with control group mean values set to 100, with error bars representing standard deviation. Statistical analysis was performed using Welch's *t*‐test. (*p < 0.05; **p < 0.01; ***p < 0.001).

To investigate the antiproliferative effects of AMH, we used A549 and H1299 cells overexpressing AMHR2. The inhibitory effect on cell proliferation was more pronounced in the presence of recombinant human AMH than in vehicle‐treated controls, and this effect occurred in a dose‐dependent manner in both cell lines (Figure 4a). Furthermore, we investigated the effect of AMH treatment on the expression of cell cycle‐related genes detected by in silico analysis using A549 cells overexpressing AMHR2. Our results revealed two distinct groups of genes: those whose expression was decreased by AMHR2 overexpression and further reduced upon AMH addition, such as *MCM7*, *PLK1* and those whose expression was decreased by AMHR2 overexpression but remained unchanged upon AMH treatment, such as *MCM4* (Figure 4b).

## DISCUSSION

This study investigated the protein expression of AMHR2 and the role of the AMH/AMHR2 signaling pathway in NSCLC. This study had four main findings: (1) AMHR2 protein expression was detected in tumor specimens from 22.4% of the patients with lung adenocarcinoma tested. (2) No cases of lung squamous cell carcinoma showed AMHR2 protein expression. (3) *EGFR* or *KRAS* mutation status and AMHR2 expression were not significantly correlated in patients with lung adenocarcinoma. (4) Activation of the AMH/AMHR2 pathway may suppress cell proliferation in NSCLC through inhibition of cell cycle‐promoting genes.

AMHR2 protein expression has been primarily studied in gynecological cancers. In the first immunohistochemical study of a large panel of epithelial ovarian cancers and other gynecological cancers, AMHR2 was detected in nearly 70% of epithelial ovarian cancers,^13^ as well as in most endometrial cancers and ovarian dysgerminomas.^13^, ^34^ Recently, AMHR2 expression in nongynecological cancers has been demonstrated in a large panel of tumor samples, showing that AMHR2 is expressed in the plasma membrane in more than 50% of the renal cell carcinomas, hepatocellular carcinomas, colorectal cancers, and NSCLC samples analyzed.^13^ However, the study included only 18 cases of NSCLC, approximately 30% of which were large cell and sarcomatoid cancers. Our data showed that AMHR2 protein expression was detected in 22.4% of lung adenocarcinoma cases and in none of the lung squamous cell carcinoma cases, which is similar to the results of a TCGA transcriptomic analysis for lung adenocarcinoma.^18^ Many questions as to why AMHR2 is expressed in some lung adenocarcinomas remain unanswered. Based on our analysis, we did not find any correlation between specific patient backgrounds, including driver oncogene mutations and AMHR2 protein expression. It has been hypothesized that reactivation of AMHR2 in tumors may occur during dedifferentiation, which is characterized by the reactivation of fetal protein expression commonly observed in carcinogenesis.^35^ Further studies on the mechanism underlying the reactivation of AMHR2 expression in lung adenocarcinoma are required.

In the present study, TCGA transcriptomic data of lung adenocarcinoma showed the upregulation of cell cycle‐promoting genes in the AMHR2‐low group. This suggests that the AMHR2‐high group exhibited cell cycle suppression, indicating slower cancer progression. The AMH‐AMHR2 signaling pathway has been reported to play a role in cell cycle regulation by inhibiting the progression of cells from the G1 to S phase,^10^, ^36^, ^37^, ^38^, ^39^ particularly in the development of the gonads and reproductive system. Based on our GSEA data, the gene set involved in the transition from the G1 to S phase was the most repressed among the cell cycle gene sets in the AMHR2‐high group.

In this study, we validated the in silico analysis results obtained from the TCGA dataset using A549 cells overexpressing AMHR2. We also examined changes in gene expression upon AMH treatment, revealing two distinct sets of genes. In A549 cells overexpressing AMHR2, we identified one set of genes, exemplified by *MCM4*, that exhibited decreased expression with AMHR2 overexpression but remained unchanged upon AMH addition. This suggests a receptor‐mediated regulation independent of ligand activation. Conversely, another group, including *MCM7* and *PLK1*, demonstrated further decreased expression upon AMH treatment, indicating a direct influence of receptor activation. These differential responses indicate relevance of the receptor signaling pathway and suggest that its activation has an inhibitory effect on the cell cycle in lung cancer cells, highlighting its potential as a therapeutic target for further investigation.

AMH binds to AMHR2 on the cell surface and activates intracellular signaling pathways that inhibit the activity of proteins necessary for cell cycle progression. AMH has been shown to inhibit the growth of human endometrial cancer cell lines with high AMHR2 expression by increasing cell cycle arrest and apoptosis.^14^, ^23^, ^38^, ^40^ Breast cancer cell growth has also been shown to be inhibited by AMH in vitro and in vivo in mice by interfering with cell cycle progression and the induction of apoptosis.^41^, ^42^ Although increasing evidence has shown that activation of the AMH‐AMHR2 pathway leads to cell cycle delays not only in gynecological cancers but also in several non‐gynecological cancers,^13^, ^43^, ^44^ it is still uncertain at what point in the cell cycle this pathway acts upon. Based on our data, the activation of the AMH/AMHR2 pathway also suppressed cell growth in NSCLC. When AMH was added to NSCLC cell lines, the inhibitory effect on cell growth was more pronounced in cells overexpressing AMHR2. These results are consistent with those observed in ovarian, endometrial, and cervical cancers, which express high AMHR2 levels.^36^, ^37^, ^40^, ^45^, ^46^ Taken together, these findings demonstrate the potential of the AMH‐AMHR2 pathway as a candidate for molecular targeted therapy; however, simply administering AMH to cancers with high AMHR2 expression may not necessarily be appropriate. Indeed, preclinical in vivo models have demonstrated the antitumor activity of murlentamab, a monoclonal antibody targeting AMHR2, suggesting that antibodies similar to this may exert antitumor effects by acting on immune cells.^13^ A deeper understanding of how the activation of this pathway regulates the cell cycle and its effect on immune cells surrounding tumors is needed.

Despite our promising findings, this study had several limitations. First, it was a single‐center study with a small number of clinical specimens. Future studies are required to determine whether consistent results can be obtained using a larger number of specimens. Furthermore, activation of the AMH‐AMHR2 pathway regulates cell cycle‐related genes in lung cancer cells; however, the mechanism of regulation remains to be elucidated. Studies on other cancer types have indicated that the mechanisms of cell cycle regulation recognized in cancer cells may be distinct from those of classical pathway regulation in reproductive organ differentiation, and further intensive studies are warranted.

## AUTHOR CONTRIBUTIONS

Yoshika Koinuma, Yoichiro Mitsuishi and Fumiyuki Takahashi: Conceptualization. Yoshika Koinuma, Yoichiro Mitsuishi, Wira Winardi, Koichiro Kanamori, Takuo Hayashi, Moulid Hidayat and Aditya Wirawan: Formal analysis. Yoshika Koinuma, Yoichiro Mitsuishi, Wira Winardi, Fumiyuki Takahashi and Kazuhisa Takahashi: Writing—original draft. Yoshika Koinuma, Yoichiro Mitsuishi, Daisuke Hayakawa, Naohisa Matsumoto, Naoko Shimada, Yoichiro Mitsuishi, Ken Tajima, Kazuya Takamochi, Fumiyuki Takahashi, Kenji Suzuki and Kazuhisa Takahashi: Writing—review and editing.

## FUNDING INFORMATION

This work was supported by JSPS KAKENHI Grant Number 15K09230 (Kazuhisa Takahashi) and Grant Number 23K07636 (Yoichiro Mitsuishi), and a grant from the Institute for Environmental and Gender‐specific Medicine, Juntendo University.

## CONFLICT OF INTEREST STATEMENT

The authors declare no conflicts of interest for this work.

## Supporting information


**Data S1.** Supporting Information.

## References

[1] Siegel RL , Miller KD , Wagle NS , Jemal A . Cancer statistics, 2023. CA Cancer J Clin. 2023;73:17–48. 36633525 10.3322/caac.21763

[2] Ferrara MG , Di Noia V , D'Argento E , et al. Oncogene‐addicted non‐small‐cell lung cancer: treatment opportunities and future perspectives. Cancers (Basel). 2020;12:1196. 32397295 10.3390/cancers12051196 PMC7281569

[3] Hanna NH , Robinson AG , Temin S , Baker S Jr , Brahmer JR , Ellis PM , et al. Therapy for stage IV non‐small‐cell lung cancer with driver alterations: ASCO and OH (CCO) joint guideline update. J Clin Oncol. 2021;39:1040–1091. 33591844 10.1200/JCO.20.03570

[4] Hanna NH , Schneider BJ , Temin S , Baker S Jr , Brahmer J , Ellis PM , et al. Therapy for stage IV non‐small‐cell lung cancer without driver alterations: ASCO and OH (CCO) joint guideline update. J Clin Oncol. 2020;38:1608–1632. 31990617 10.1200/JCO.19.03022

[5] Massagué J , Blain SW , Lo RS . TGFβ signaling in growth control, cancer, and heritable disorders. Cell. 2000;103:295–309. 11057902 10.1016/s0092-8674(00)00121-5

[6] Howard JA , Hart KN , Thompson TB . Molecular mechanisms of AMH signaling. Front Endocrinol (Lausanne). 2022;13:927824. 35813657 10.3389/fendo.2022.927824 PMC9256959

[7] Mishina Y , Whitworth DJ , Racine C , Behringer RR . High specificity of Müllerian‐inhibiting substance signaling in vivo. Endocrinology. 1999;140:2084–2088. 10218958 10.1210/endo.140.5.6705

[8] Behringer RR , Finegold MJ , Cate RL . Müllerian‐inhibiting substance function during mammalian sexual development. Cell. 1994;79:415–425. 7954809 10.1016/0092-8674(94)90251-8

[9] Durlinger ALL , Visser JA , Themmen APN . Regulation of ovarian function: the role of anti‐Müllerian hormone. Reproduction. 2002;124:601–609. 12416998 10.1530/rep.0.1240601

[10] Ha TU , Segev DL , Barbie D , Masiakos PT , Tran TT , Dombkowski D , et al. Mullerian inhibiting substance inhibits ovarian cell growth through an Rb‐independent mechanism. J Biol Chem. 2000;275:37101–37109. 10958795 10.1074/jbc.M005701200

[11] Teixeira J , Maheswaran S , Donahoe PK . Mullerian inhibiting substance: an instructive developmental hormone with diagnostic and possible therapeutic applications. Endocr Rev. 2001;22:657–674. 11588147 10.1210/edrv.22.5.0445

[12] Chauvin M , Garambois V , Colombo P‐E , Chentouf M , Gros L , Brouillet JP , et al. Anti‐Mullerian hormone (AMH) autocrine signaling promotes survival and proliferation of ovarian cancer cells. Sci Rep. 2021;11:2231. 33500516 10.1038/s41598-021-81819-y PMC7838181

[13] Barret J‐M , Nicolas A , Jarry A , et al. The expression of anti‐Mullerian hormone type II receptor (AMHRII) in non‐gynecological solid tumors offers potential for broad therapeutic intervention in cancer. Biology (Basel). 2021;10:305. 33917111 10.3390/biology10040305 PMC8067808

[14] Gowkielewicz M , Lipka A , Piotrowska A , Szadurska‐Noga M , Nowakowski J , Dzięgiel P , et al. Anti‐Müllerian hormone expression in endometrial cancer tissue. Int J Mol Sci. 2019;20:1325. 30884769 10.3390/ijms20061325 PMC6471522

[15] La Marca A , Volpe A . The anti‐Mullerian hormone and ovarian cancer. Hum Reprod Update. 2007;13:265–273. 17213257 10.1093/humupd/dml060

[16] Kersual N , Garambois V , Chardès T , Pouget JP , Salhi I , Bascoul‐Mollevi C , et al. The human Mullerian inhibiting substance type II receptor as immunotherapy target for ovarian cancer. Validation using the mAb 12G4. MAbs. 2014;6:1314–1326. 25517316 10.4161/mabs.29316 PMC4623115

[17] Park SH , Chung YJ , Song JY , Kim SI , Pépin D , MacLaughlin DT , et al. Müllerian inhibiting substance inhibits an ovarian cancer cell line via β‐catenin interacting protein deregulation of the Wnt signal pathway. Int J Oncol. 2017;50:1022–1028. 28197641 10.3892/ijo.2017.3874

[18] Beck TN , Korobeynikov VA , Kudinov AE , Georgopoulos R , Solanki NR , Andrews‐Hoke M , et al. Anti‐Mullerian hormone signaling regulates epithelial plasticity and chemoresistance in lung cancer. Cell Rep. 2016;16:657–671. 27396341 10.1016/j.celrep.2016.06.043 PMC4956518

[19] Cancer Genome Atlas Research Network , Weinstein JN , Collisson EA , et al. The cancer genome atlas pan‐cancer analysis project. Nat Genet. 2013;45:1113–1120. 24071849 10.1038/ng.2764 PMC3919969

[20] Goldstraw P , Crowley J , Chansky K , Giroux DJ , Groome PA , Rami‐Porta R , et al. The IASLC lung cancer staging project: proposals for the revision of the TNM stage groupings in the forthcoming (seventh) edition of the TNM classification of malignant tumours. J Thorac Oncol. 2007;2:706–714. 17762336 10.1097/JTO.0b013e31812f3c1a

[21] Edge SB , Compton CC . The American joint committee on cancer: the 7th edition of the AJCC cancer staging manual and the future of TNM. Ann Surg Oncol. 2010;17:1471–1474. 20180029 10.1245/s10434-010-0985-4

[22] Barretina J , Caponigro G , Stransky N , Venkatesan K , Margolin AA , Kim S , et al. Addendum: the cancer cell line encyclopedia enables predictive modelling of anticancer drug sensitivity. Nature. 2019;565:E5–E6. 30559381 10.1038/s41586-018-0722-x

[23] Gowkielewicz M , Lipka A , Majewska M , Piotrowska A , Szadurska‐Noga M , Nowakowski JJ , et al. Anti‐Mullerian hormone type II receptor expression in endometrial cancer tissue. Cells. 2020;9:2312. 33080800 10.3390/cells9102312 PMC7603004

[24] Love MI , Huber W , Anders S . Moderated estimation of fold change and dispersion for RNA‐seq data with DESeq2. Genome Biol. 2014;15:550. 25516281 10.1186/s13059-014-0550-8 PMC4302049

[25] Raudvere U , Kolberg L , Kuzmin I , Arak T , Adler P , Peterson H , et al. G:profiler: a web server for functional enrichment analysis and conversions of gene lists (2019 update). Nucleic Acids Res. 2019;47:W191–W198. 31066453 10.1093/nar/gkz369 PMC6602461

[26] Reimand J , Kull M , Peterson H , Hansen J , Vilo J . G:profiler—a web‐based toolset for functional profiling of gene lists from large‐scale experiments. Nucleic Acids Res. 2007;35:W193–W200. 17478515 10.1093/nar/gkm226 PMC1933153

[27] Subramanian A , Tamayo P , Mootha VK , Mukherjee S , Ebert BL , Gillette MA , et al. Gene set enrichment analysis: a knowledge‐based approach for interpreting genome‐wide expression profiles. Proc Natl Acad Sci U S A. 2005;102:15545–15550. 16199517 10.1073/pnas.0506580102 PMC1239896

[28] GTEx Consortium . The genotype‐tissue expression (GTEx) project. Nat Genet. 2013;45:580–585. 23715323 10.1038/ng.2653 PMC4010069

[29] Song JY , Chen KY , Kim SY , Kim MR , Ryu KS , Cha JH , et al. The expression of Mullerian inhibiting substance/anti‐Mullerian hormone type II receptor protein and mRNA in benign, borderline and malignant ovarian neoplasia. Int J Oncol. 2009;34:1583–1591. 19424576 10.3892/ijo_00000288

[30] de Kat AC , van der Schouw YT , Eijkemans MJC , Herber‐Gast GC , Visser JA , Verschuren WMM , et al. Back to the basics of ovarian aging: a population‐based study on longitudinal anti‐Müllerian hormone decline. BMC Med. 2016;14:151. 27716302 10.1186/s12916-016-0699-y PMC5046975

[31] Kanehisa M . Toward understanding the origin and evolution of cellular organisms. Protein Sci. 2019;28:1947–1951. 31441146 10.1002/pro.3715 PMC6798127

[32] Kanehisa M , Goto S . KEGG: kyoto encyclopedia of genes and genomes. Nucleic Acids Res. 2000;28:27–30. 10592173 10.1093/nar/28.1.27 PMC102409

[33] Kanehisa M , Furumichi M , Sato Y , Kawashima M , Ishiguro‐Watanabe M . KEGG for taxonomy‐based analysis of pathways and genomes. Nucleic Acids Res. 2023;51:D587–D592. 36300620 10.1093/nar/gkac963 PMC9825424

[34] Bakkum‐Gamez JN , Aletti G , Lewis KA , Keeney GL , Thomas BM , Navarro‐Teulon I , et al. Mullerian inhibiting substance type II receptor (MISIIR): a novel, tissue‐specific target expressed by gynecologic cancers. Gynecol Oncol. 2008;108:141–148. 17988723 10.1016/j.ygyno.2007.09.010

[35] Tanwar PS , Commandeur AE , Zhang L , Taketo MM , Teixeira JM . The Müllerian inhibiting substance type 2 receptor suppresses tumorigenesis in testes with sustained β‐catenin signaling. Carcinogenesis. 2012;33:2351–2361. 22962306 10.1093/carcin/bgs281 PMC3510735

[36] Song JY , Jo HH , Kim MR , Lew YO , Ryu KS , Cha JH , et al. Expression of Mullerian inhibiting substance type II receptor and antiproliferative effects of MIS on human cervical cancer. Int J Oncol. 2012;40:2013–2021. 22344630 10.3892/ijo.2012.1370 PMC5609185

[37] Hwang SJ , Suh MJ , Yoon JH , et al. Identification of characteristic molecular signature of Mullerian inhibiting substance in human HPV‐related cervical cancer cells. Int J Oncol. 2011;39:811–820. 21573503 10.3892/ijo.2011.1042 PMC5609187

[38] Kim SI , Yoon JH , Hur SY . Functional profiles of Mullerian inhibiting substance/anti‐Mullerian hormone (MIS/AMH) in primarily cultured endometrial cancer cells. J Cancer. 2021;12:6289–6300. 34539902 10.7150/jca.60700 PMC8425195

[39] Wang J , Dicken C , Lustbader JW , Tortoriello DV . Evidence for a Mullerian‐inhibiting substance autocrine/paracrine system in adult human endometrium. Fertil Steril. 2009;91:1195–1203. 18328480 10.1016/j.fertnstert.2008.01.028

[40] Zhang T , Deng L , Xiong Q , Su S , Gu J . Anti‐Mullerian hormone inhibits proliferation and induces apoptosis in epithelial ovarian cancer cells by regulating the cell cycle and decreasing the secretion of stem cell factor. Oncol Lett. 2018;16:3260–3266. 30127923 10.3892/ol.2018.8985 PMC6096103

[41] Segev DL , Ha TU , Tran TT , Kenneally M , Harkin P , Jung M , et al. Mullerian inhibiting substance inhibits breast cancer cell growth through an NFkappa B‐mediated pathway. J Biol Chem. 2000;275:28371–28379. 10874041 10.1074/jbc.M004554200

[42] Gupta V , Yeo G , Kawakubo H , Rangnekar V , Ramaswamy P , Hayashida T , et al. Mullerian‐inhibiting substance induces Gro‐beta expression in breast cancer cells through a nuclear factor‐kappaB‐dependent and Smad1‐dependent mechanism. Cancer Res. 2007;67:2747–2756. 17363596 10.1158/0008-5472.CAN-06-2312

[43] Hoshiya Y , Gupta V , Segev DL , Hoshiya M , Carey JL , Sasur LM , et al. Mullerian inhibiting substance induces NFkB signaling in breast and prostate cancer cells. Mol Cell Endocrinol. 2003;211:43–49. 14656475 10.1016/j.mce.2003.09.010

[44] Chauvin M , Meinsohn M‐C , Dasari S , May P , Iyer S , Nguyen NMP , et al. Cancer‐associated mesothelial cells are regulated by the anti‐Mullerian hormone axis. Cell Rep. 2023;42:112730. 37453057 10.1016/j.celrep.2023.112730

[45] Stephen AE , Pearsall LA , Christian BP , Donahoe PK , Vacanti JP , MacLaughlin DT . Highly purified müllerian inhibiting substance inhibits human ovarian cancer in vivo. Clin Cancer Res. 2002;8:2640–2646. 12171896

[46] MacLaughlin DT , Donahoe PK . Müllerian inhibiting substance/anti‐ Müllerian hormone: a potential therapeutic agent for human ovarian and other cancers. Future Oncol. 2010;6:391–405. 20222796 10.2217/fon.09.172 PMC3935316

