# Supplementary material for: Anti‐Müllerian hormone type II receptor protein expression in non‐small cell lung cancer and the effect of AMH/AMHR2 signaling on cancer cell proliferation
Source: Thorac Cancer. 2024 Sep 4;15(29):2090–9. doi: 10.1111/1759-7714.15309 (PMC11471453; doi:10.1111/1759-7714.15309)
Supplement: Supplementary file 1 — Data S1. Supporting Information. [file TCA-15-2090-s001.docx]

**Supplementary Figure 1: Comparative analysis of AMHR2 expression across various normal tissues using data sourced from the Genotype-Tissue Expression (GTEx) database.**

Violin plots showing the distribution of AMHR2 expression in each tissue type. Notably, reproductive organs, such as the ovaries, exhibit high AMHR2 expression, whereas normal lung tissues show lower expression. GTEx Analysis Release V8 (dbGaP Accession phs000424.v8.p2) on November 8^th^, 2023.

**Supplementary Figure 2: Comparative analysis of AMHR2 expression across various cancer tissues using data sourced from TCGA database.**

Scatter plots showing the distribution of AMHR2 mRNA expression in each tumor type. Notably, AMHR2 is highly expressed in tumors other than those originating from the Müllerian duct, such as adrenocortical carcinoma, cutaneous melanoma, pheochromocytoma, malignant pleural mesothelioma, and lung adenocarcinoma. Expression was quantified using RSEM (batch-normalized to Illumina HiSeq_RNASeqV2).

Abbreviations:

LAML: Acute Myeloid Leukemia

ACC: Adrenocortical Carcinoma

LGG: Lower Grade Glioma

BLCA: Bladder Urothelial Carcinoma

BRCA: Breast Invasive Ductal Carcinoma

CESC: Cervical Squamous Cell Carcinoma and Endocervical Adenocarcinoma

SKCM: Cutaneous Melanoma

DLBC: Diffuse Large B-Cell Lymphoma

ESCA: Esophageal Carcinoma

GBM: Glioblastoma Multiforme

HNSC: Head and Neck Squamous Cell Carcinoma

LIHC: Hepatocellular Carcinoma

LUAD: Lung Adenocarcinoma

LUSC: Lung Squamous Cell Carcinoma

TGCT: Mixed Germ Cell Tumor

PAAD: Pancreatic Adenocarcinoma

KIRP: Papillary Renal Cell Carcinoma

THCA: Papillary Thyroid Cancer

PGL: Paraganglioma

PCPG: Pheochromocytoma

MESO: Pleural Mesothelioma

PRAD: Prostate Adenocarcinoma

KIRC: Renal Clear Cell Carcinoma

SEMC: Seminoma

OVAR: Serous Ovarian Cancer

THYM: Thymoma

UCS: Uterine Carcinoma

UCEC: Uterine Endometrioid Carcinoma

**Supplementary Figure 3: Comparative analysis of AMHR2 mRNA expression across various cancer types in the Cancer Cell Line Encyclopedia database.**

Box plots showing the distribution of AMHR2 expression in each cancer type. Non-small cell lung cancer cell lines show lower AMHR2 expression than many other cancer types. The A549 and H1299 cell lines used in our experiments were characterized by low AMHR2 expression.

**Supplementary Figure 4: Pathway enrichment analysis of the significantly downregulated genes in the AMHR2-low group.**

The cluster lists for KEGG (upper) and Biological Process (lower) are shown.

**Supplementary Figure 5: Gene set enrichment analysis showing the gene clusters that positively promote the cell cycle.**

This figure highlights the results for the gene groups associated with cell cycle progression in relation to those presented in Figure 2c.
